# Anthropogenic noise changes arthropod abundances

**DOI:** 10.1002/ece3.2698

**Published:** 2017-03-23

**Authors:** Jessie P. Bunkley, Christopher J. W. McClure, Akito Y. Kawahara, Clinton D. Francis, Jesse R. Barber

**Affiliations:** ^1^ Department of Biological SciencesBoise State UniversityBoiseIDUSA; ^2^ The Peregrine FundBoiseIDUSA; ^3^ Florida Museum of Natural HistoryUniversity of FloridaGainesvilleFLUSA; ^4^ Department of Biological SciencesCalifornia Polytechnic State UniversitySan Luis ObispoCAUSA

**Keywords:** compressors, energy extraction, insects, invertebrates, natural gas, noise pollution

## Abstract

Anthropogenic noise is a widespread and growing form of sensory pollution associated with the expansion of human infrastructure. One specific source of constant and intense noise is that produced by compressors used for the extraction and transportation of natural gas. Terrestrial arthropods play a central role in many ecosystems, and given that numerous species rely upon airborne sounds and substrate‐borne vibrations in their life histories, we predicted that increased background sound levels or the presence of compressor noise would influence their distributions. In the second largest natural gas field in the United States (San Juan Basin, New Mexico, USA), we assessed differences in the abundances of terrestrial arthropod families and community structure as a function of compressor noise and background sound level. Using pitfall traps, we simultaneously sampled five sites adjacent to well pads that possessed operating compressors, and five alternate, quieter well pad sites that lacked compressors, but were otherwise similar. We found a negative association between sites with compressor noise or higher levels of background sound and the abundance of five arthropod families and one genus, a positive relationship between loud sites and the abundance of one family, and no relationship between noise level or compressor presence and abundance for six families and two genera. Despite these changes, we found no evidence of community turnover as a function of background sound level or site type (compressor and noncompressor). Our results indicate that anthropogenic noise differentially affects the abundances of some arthropod families. These preliminary findings point to a need to determine the direct and indirect mechanisms driving these observed responses. Given the diverse and important ecological functions provided by arthropods, changes in abundances could have ecological implications. Therefore, we recommend the consideration of arthropods in the environmental assessment of noise‐producing infrastructure.

## 1 Introduction

Anthropogenic noise has been shown to alter the behavior and distribution of animals in aquatic (Nowacek, Thorne, Johnston, & Tyack, 2007; Slabbekoorn et al., 2010) and terrestrial (Francis & Barber, 2013) environments. Vehicular traffic, urbanization, and energy extraction infrastructure are widespread sources of this sensory pollutant and increase the background sound levels of many ecosystems (Barber, Crooks, & Fristrup, 2010; Barber et al., 2011). Compressors associated with the extraction and transportation of natural gas could be a source of disturbance because they are widespread and produce chronic, broadband noise (Francis & Barber, 2013). Studies examining anthropogenic noise at a landscape scale have focused on the responses of vertebrates (for reviews, see, Barber et al., 2010; Francis & Barber, 2013; Nowacek et al., 2007; Slabbekoorn et al., 2010); yet, there have not been investigations into possible impacts on arthropod distributions or community structure.

Arthropods are a critical component of food webs and provide many ecosystem functions including pollination, seed dispersal, herbivory, decomposition, and habitat formation (Prather et al., 2012). Given their fundamental role in many ecosystems, it is essential to understand the potential effects of anthropogenic noise on arthropods and other invertebrates (Morley, Jones, & Radford, 2014). Arthropods use sound for a variety of purposes, including the detection of predators and prey, and for intraspecific communication. Previous research demonstrates that some arthropods are affected by loud anthropogenic infrastructure (Morley et al., 2014); for instance, bow‐winged grasshoppers, *Chorthippus biguttulus* (Orthoptera: Acrididae), found near loud roadside sites produce higher frequency calls than individuals from quiet sites (Lampe, Schmoll, Franzke, & Reinhold, 2012); the cicada species, *Cryptotympana takasagona* (Hemiptera: Cicadidae), exhibits a strong positive correlation between call frequency and noise exposure level in urban parks (Shieh, Liang, Chen, Loa, & Liao, 2012); and in traffic noise, female field crickets, *Gryllus bimaculatus* (Orthoptera: Gryllidae), fail to orient to played‐back male calls (Schmidt, Morrison, & Kunc, 2014). Furthermore, studies have also documented changes in the activity levels and distributions of arthropod predators, such as bat and bird communities, as a response to louder soundscapes and compressor noise specifically (Bunkley, McClure, Kleist, Francis, & Barber, 2015; Bayne et al., 2008; Francis, Ortega, & Cruz, 2009). Anthropogenic noise, therefore, may affect arthropods both directly, through disrupted communication and environmental perception, and indirectly, via complex trophic interactions.

Here we investigate two possible hypotheses of how terrestrial arthropod abundances and community structure at the landscape scale might be impacted or altered by anthropogenic noise (1) in a dose–response fashion from elevated background sound levels or (2) by the presence of persistent, intense, and broadband gas compressor noise alone, independent of overall sound level. We predicted that anthropogenic noise would negatively affect terrestrial arthropods that are known to use sound and/or vibratory stimuli (Table 1).

**Table 1:** Taxonomic grouping of arthropods with more than 10 specimens collected, reference(s) indicating use of airborne or substrate‐borne vibrations, numbers of specimens collected at noncompressor and compressor sites, and the observed response to noise (significance is denoted with an *). Genera were tested only if a response to noise (compressor or dB) was observed at the family level

Order	Family	Genus	References indicating use of airborne or substrate‐borne vibrations	Specimens from Noncompressor Sites	Specimens from Compressor Sites	Total Number of Specimens	Response to Noise
Araneae			Greenfield (2002)				
	Salticidae (jumping spider)		Shamble et al. (2016)	9	6	15	None
		*Habrocestum*		8	6	14	Not tested
	Gnaphosidae (ground spider)			40	70	110	None
		*Callilepis*		5	15	20	Not tested
		*Micaria*		21	34	55	Not tested
	Lycosidae (wolf spider)			13	5	18	dB−*
		*Pardosa*		10	3	13	dB−*
Coleoptera	Chrysomelidae (leaf beetle)		Drosopoulos & Claridge (2006), Greenfield (2002)	6	7	13	None
	Tenebrionidae (darkling beetle)	* *	Drosopoulos & Claridge (2006), Greenfield, (2002)	10	16	26	None
		*Eleodes*		7	9	16	Not tested
		*Periscepsia*		9	5	14	Not tested
Hemiptera	Cercopidae (froghoppers)		Drosopoulos & Claridge (2006)	16	5	21	Compressor−*
	Cicadellidae (leafhoppers)		Drosopoulos & Claridge (2006), Hoy & Robert (1996)	64	87	151	dB+*
Hymenoptera	Formicidae (ants)		Drosopoulos & Claridge (2006), Greenfield (2002)	304	477	781	None
		*Camponotus*		67	78	145	Not tested
		*Crematogaster*		27	4	31	Not tested
		*Forelius*		12	38	50	Not tested
		*Formica*		17	23	40	Not tested
		*Liometopum*		2	12	14	Not tested
		*Myrmecocystus*		148	306	454	Not tested
		*Pogonomyrmex*		15	1	16	Not tested
	Mutillidae (velvet ants)	* *	Polidori et al. (2013)	10	3	13	dB−*
Orthoptera	Gryllidae (crickets)	*Gryllus*	Drosopoulos & Claridge (2006), Greenfield (2002), Hedwig (2014), Hoy & Robert (1996)	8	6	14	None
	Acrididae (grasshoppers)		Drosopoulos & Claridge (2006), Greenfield (2002), Hedwig (2014), Hoy & Robert (1996)	60	37	97	Compressor−*
		*Xanthippus*		12	8	20	None
		*Spharagemon*		11	9	20	None
	Rhaphidophoridae (cave, camel, and spider crickets)	*Ceuthophilus (camel crickets)*	Drosopoulos & Claridge (2006), Greenfield (2002)	41	1	42	Compressor−*

## 2 Methods

We conducted this study in the Rattlesnake Canyon Habitat Management Area, in the San Juan Basin of northwestern New Mexico, from May to June 2013. This is the second largest gas basin in the United States and produces an estimated one trillion cubic feet of gas per year (Fassett, 2010). This region has a long history of resource extraction, beginning with the discovery of natural gas in 1921 and a marked increase in extraction efforts during the 1950s (Fassett, 2010). Consequently, this landscape has experienced over 60 years of intensive disturbance, including extensive noise pollution. Plant communities in this arid region are dominated by piñon pine (*Pinus edulis*) and Utah juniper (*Juniperus osteosperma*), with components of sagebrush (*Artemisia tridentata*) and open grassland (Francis et al., 2009).

Within the gas field, we simultaneously sampled five separate sites adjacent to well pads with gas compressors (compressor) and five different sites next to well pads without compressors (noncompressor). Compressor and noncompressor sites were at least 0.5 km apart to ensure different acoustic conditions (see Appendix S1). Due to the high density of compressors on the landscape, noncompressor site sound levels were likely still influenced by distant compressor stations. Moreover, as a result of intensive gas extraction in this region, background sound levels were elevated at all sites. Although some compressor and noncompressor sites had similar background sound levels (see Appendix S2), the composition of those background sounds was different, with compressor sites being dominated by characteristic compressor noise and noncompressor sites having other forms of noise, such as water pump jacks. Compressor and noncompressor sites did not vary in vegetative characteristics for canopy cover, bare ground, live matter, rock, dead wood, grasses and forbs, litter depth, live and dead juniper and piñon trees, shrubs, and total trees (Francis et al., 2009); however, recruitment of piñon trees may slowly be changing between compressor and noncompressor sites (Francis, Kleist, Ortega, & Cruz, 2012) and differences may be present at smaller scales. All sites lacked artificial illumination at night, controlling for effects of light pollution on arthropod communities (Davies, Bennie, & Gaston, 2012). This design, in addition to temporally simultaneous sampling, helped to isolate noise as the variable of interest by controlling for moon phase, presence of roads and infrastructure, and edge effects.

We sampled the terrestrial arthropod community with passive‐capture pitfall traps and compared the relative abundance of specimens between sites (Spence & Niemela, 1994). To construct pitfall traps, we used clear 500‐mL plastic containers buried flush with the ground and partially filled with 100% ethanol and ran strips of white plastic (0.5 m × 3 m) vertically across the ground as guide vanes for directing walking arthropods toward the trap opening, thus attempting to increase the number of specimens captured. Because this sampling method is designed for terrestrial arthropods and is not appropriate for sampling aerial arthropods, any incidentally collected specimens from taxa whose primary mode of locomotion is flight were excluded from analyses; this included Diptera, Hymenoptera (excluding Formicidae and Mutillidae), Lepidoptera, and Neuroptera (McIntyre, Rango, Fagan, & Faeth, 2001). At each site, we arranged four traps in an “X” grid, 50 m away from the compressor or the center of the noncompressor site (see Appendix S3). We checked trap contents every other day for 2 weeks, resulting in seven collection events for each site, and subsequently identified the collected arthropods to the level of family and, when possible, to genus.

At each site, we installed Acoustic Recording Units (ARUs; Roland R05 or R09; MP3 128 kbps) 50 m away from the center of the site to record the background sound level at the pitfall trap distance for at least 3 days during the trapping period (Mennitt & Fristrup, 2012). We used custom programs (Damon Joyce, NPS, AUDIO2NVSPL) to convert the MP3 recordings into hourly sound pressure levels and then to hourly LEQ (equivalent continuous sound level) values in dB(A) (Damon Joyce, NPS, Acoustic Monitoring Toolbox). These hourly sound levels were averaged over the duration of the ARU deployment, which allowed us to use the continuous variable of background sound level (dBA) in statistical analyses (see Appendix S3). Importantly, these background sound levels are a composite of all sounds in the environment, including natural and anthropogenic sources, such as wind, rain, water pumps, and compressors. Due to the high density of compressors in the region, some amount of compressor noise is likely present at some of the noncompressor sites; however, at compressor sites, this characteristic noise dominated the background sound level.

We analyzed the effects of noise on the abundance of those arthropod families for which we collected at least 10 total specimens (Davies et al., 2012; Gotelli & Ellison, 2004), which allowed enough variation for analyses, using Poisson‐distributed generalized linear mixed‐effects models with random intercepts for site for the repeated sampling of each two night period of trap deployment (program R [R Core Development Team, 2015], CRAN packages: lme4, MASS, and psych). We tested our two a priori hypotheses that louder soundscapes in general and compressor noise specifically affect arthropod abundances by building models that either contained site‐specific dB(A) levels or the discrete compressor/noncompressor variable. We tested these biological hypotheses by ranking and comparing these two models—dB and compressor—as well as a null model using Akaike's information criterion (Akaike, 1974). This analytical approach is employed for hypothesis testing and not parameter estimation. If either the dB or compressor models received a lower AIC value than the null model, we considered that hypothesis to be supported marginally or strongly if the 85% or 95% confidence intervals respectively excluded zero, indicating that these noise variables are informative, regardless of other models that might fall within ∆2 AIC (Arnold, 2010). In this system, the 2 days between sampling events were considered sufficient for terrestrial arthropod communities to move and mix randomly (see Appendix S4). Therefore, the sample size was 70 (seven sampling occasions for each of the 10 sites) for all families in our study, and each model contained a random intercept for site to control for the repeated sampling design. For those families where one of the noise variables was an informative parameter, we performed separate analyses on nested genera (if there was more than one).

We also evaluated the community‐level responses to noise using the full family dataset (i.e., including families with <10 individuals). We used Welch two‐sample *t*‐tests to compare the rarefied family richness (minimum sample of 54 individuals; program R, CRAN packages: VEGAN, rarefy) and Chao1 asymptotic richness estimators (program R, CRAN packages: VEGAN, estimateR) between compressor and noncompressor sites (program R, CRAN; Gotelli & Colwell, 2011). To gauge sampling completeness, we generated rarefaction curves for each site (program R, CRAN package: VEGAN; Gotelli & Colwell, 2001; Rarefaction function: Jacobs, 2011). Using a permutational analysis of variance (PERMANOVA), we tested whether the compressor factor or background dB level caused differences in the Bray–Curtis or Cao dissimilarity matrices calculated from abundance data at the family and genus levels (program R, CRAN package: VEGAN). Families or genera that only occurred at one site were excluded from PERMANOVA analyses (Ohwaki, 2015), and for the Bray–Curtis analyses the abundance data were square‐root‐transformed to reduce leverage by dominant taxa (program R, CRAN package: VEGAN; Davies et al., 2012). Finally, nonmetric multidimensional scaling (NMDS) was used to visualize the relationships of the sites with one another using the full datasets and Bray–Curtis dissimilarity.

## 3 Results

We collected 1,771 arthropod specimens and identified 96% to family and 72% to genus. The best model for two insect families, leafhoppers (Hemiptera: Cicadellidae) and velvet ants (Hymenoptera: Mutillidae), and the wolf spider family (Araneae: Lycosidae) and genus (Lycosidae: *Pardosa*), included a covariate for sound level (dBA). The top model for three insect families—grasshoppers (Orthoptera: Acrididae), cave, camel, and spider crickets (Orthoptera: Rhaphidophoridae, *Ceuthophilus*), and froghoppers (Hemiptera: Cercopidae)—included a factor for compressor (see Appendix S5). The null model was the top model for six families and two genera (see Appendix S6), suggesting no effect of overall sound level or compressor noise specifically on these taxa.

**Figure 1:**
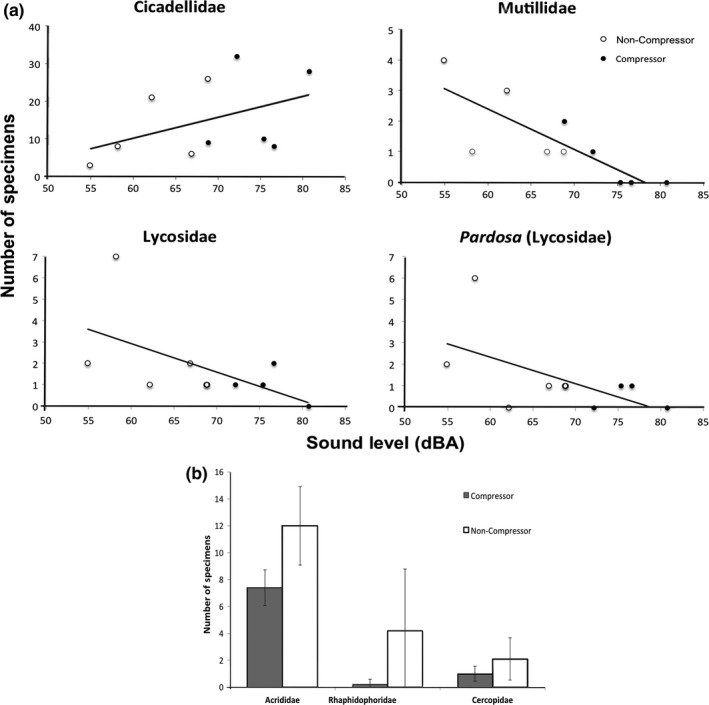
(a) The abundance of the family Cicadellidae increased as a function of background sound level (dBA), while the abundances of families Mutillidae and Lycosidae and genus *Pardosa* (Lycosidae) decreased. (b) The abundance (mean ± SE) of families Acrididae, Rhaphidophoridae, and Cercopidae exhibited a negative effect from the compressor noise factor. Gray bars represent sites with compressor noise, and white bars are noncompressor sites

The leafhopper family (Cicadellidae) was positively associated with background sound level, while the velvet ant family (Mutillidae) and wolf spider family (Lycosidae) and genus (*Pardosa*) exhibited a negative relationship, indicating these groups respond to louder soundscapes in general. For every increase of 10 dB(A), the abundance of leafhoppers (Cicadellidae) increased 44% (95% CI: 0.95–2.17), while the abundance decreased 56% (95% CI: 0.24–0.80) for velvet ants (Mutillidae), 44% (95% CI: 0.33–0.94) for the wolf spider family (Lycosidae), and 53% (95% CI: 0.46–0.48) for the wolf spider genus (*Pardosa*) (Figure 1a). All families associated with the compressor factor were negatively related, illustrating a negative response to compressor noise specifically. At sites with compressor noise, grasshoppers (Acrididae) were 24% less abundant (0.63; 95% CI: 0.36‐1.10), froghoppers (Cercopidae) were 52% less abundant (0.31; 85% CI: 0.09‐1.06), and cave, camel, and spider crickets (Rhaphidophoridae; *Ceuthophilus*) were 95% less abundant (0.03; 95% CI: 0.00–0.54; Figure 1b).

The results of the rarefaction curves indicate that sampling was incomplete for all sites (Appendix S7); therefore, we made richness comparisons between compressor and noncompressor sites in two ways: first, by generating the statistical expectation for the number of families per 54 individuals sampled per site (i.e. rarefied to the sample size of the site with the fewest individuals sampled), and second by using asymptotic richness estimators to provide a minimum family richness estimate per site (Gotelli & Colwell, 2001). Both rarefied family richness and Chao1 asymptotic richness estimates did not statistically differ between compressor sites and noncompressor sites (observed family richness: *t* = 1.50, *df* = 7.62, *p* = 0.17; asymptotic richness estimates: *t* = 0.38, *df* = 6.75, *p* = 0.72). For the analyses of families and genera, the PERMANOVA results show nonsignificant effects of the compressor factor (family: *F* = 1.15, *df* = 1, *p* = 0.39; genus: *F* = 0.76, *df* = 1, *p* = 0.67) and background dB level (family: *F* = 0.84, *df* = 1, *p* = 0.64; genus: *F* = 0.70, *df* = 1, *p* = 0.80) on the Bray–Curtis dissimilarity matrix calculated from the abundance data. Similar nonsignificant community turnover was observed for compressor (family: *F* = 1.45, *df* = 1, *p* = 0.18; genus: *F* = 1.00, *df* = 1, *p* = 0.44) and background dB level (family: *F* = 0.98, *df* = 1, *p* = 0.52; genus: *F* = 0.74, *df* = 1, *p* = 0.81) on the Cao dissimilarity matrix. Finally, the NMDS plot illustrates the grouping of compressor and noncompressor sites with a slight overlap (stress = 0.18; Appendix S8), and a post hoc fit of the background dB(A) environmental vector onto the ordination supports the relationship between the compressor designation and background dB level.

## 4 Discussion

Our findings indicate that louder background sound levels in general and broadband, chronic compressor noise, specifically, differentially affect the abundances of some arthropod families. Those groups that responded to louder (dB) demonstrate a dose–response to noise while arthropods that reacted to the binary compressor factor indicate impacts of broadband, chronic compressor noise specifically. Of those groups affected all but one were less abundant at louder sites or sites with compressor stations. This preliminary evidence prompts further exploration into why some arthropods are affected by noise, the mechanisms behind these responses, and potential ecological repercussions.

Compressor noise is broadband and has substantial energy at low frequencies, likely producing substrate‐borne vibrations (~20–5,000 Hz ± 55 dB; however, this noise likely produces lower frequencies that are not captured in this measurement due to the falloff of the frequency response of the microphone at 20 Hz; see Appendix S9). It is possible, therefore, that compressor noise or higher levels of background noise directly interfere with or mask important information used by acoustically and vibrationally sensitive arthropod taxa. Anthropogenic noise reduces the activity of some bat species (Bunkley et al., 2015) and alters bird communities (Francis et al., 2009), both of which may prey upon terrestrial arthropods. Therefore, anthropogenic noise could indirectly affect terrestrial arthropod abundances via trophic interactions. This study is unable to distinguish between direct and indirect effects on arthropod abundances; however, we cautiously explore some possible causes for these observed responses. Future studies should attempt to discriminate between direct and indirect effects of noise pollution on arthropods by experimentally applying noise treatments on a landscape scale.

All arthropod groups in our study that responded to sites with louder background sound levels or compressor noise have members that are known to produce, perceive, or use airborne or substrate‐borne vibrations. Grasshoppers (Acrididae) have tympana for receiving airborne sound and use stridulations (5–15 kHz and 20–40 kHz) for communication (Meyer & Elsner, 1996). Vibratory or seismic signals are important components of courtship communication for wolf spiders (Lycosidae; Gibson & Uetz, 2008). Jumping spiders can detect airborne sounds at distances reaching the far field, despite their lack of tympana (Shamble et al., 2016), revealing that some terrestrial arthropods may be more sensitive to distant airborne signals than previously thought. For the ground‐dwelling, or brush‐legged, wolf spider, *Schizocosa ocreata*, female receptivity and mating success are reduced when exposed to airborne white noise (Gordon & Uetz, 2012). Some cave, camel, and spider crickets (Rhaphidophoridae) use very low frequency, substrate‐borne vibrations for sexual communication (Stritih & Cokl, 2012), and velvet ants (Mutillidae) also produce low‐frequency sounds via stridulation (4–18 kHz) (Polidori, Pavan, Ruffato, Asis, & Tormos, 2013). Additionally, vibrational communication signals that indicate distress and territoriality have been recorded in several froghopper species (Cercopidae; Tishechkin, 2003). For these groups, it is possible that noise directly disrupts important signals and therefore potentially decreases fitness via reduced reproductive or foraging success or alters habitat selection decisions, resulting in distributional changes across the landscape. Leafhoppers (Cicadellidae) are also sensitive to sound (Drosopoulos & Claridge, 2006), and locate and recognize mates solely through the use of vibrational communication signals (Claridge, 1985). The counterintuitive positive relationship of leafhopper abundance with louder background sound levels (dB) might be caused by an indirect effect of trophic interactions that possibly outweigh potential direct effects of signal disruption. For leafhoppers that experience predation by vertebrate species, like Brazilian free‐tailed bats (*Tadarida brasiliensis*; Lee & McCracken, 2005), loud sites may serve as a refuge (Bunkley et al., 2015) by acting as a predator shield (sensu Berger, 2007). An alternative explanation for the positive response of leafhopper abundance to noise is the possibility for high levels of noise to act as a sensory trap. Acoustic devices have long been used to lure various insect species to traps (Mankin, 2012), and it is possible that acoustically sensitive leafhoppers (Cicadellidae) are attracted to louder sites (Saxena & Kumar, 1980). With many complex interactions potentially at play for the responses of all the arthropod families, it is clear that additional research is required to fully understand these results.

Interestingly, some arthropod groups in our dataset that are known to use sound did not exhibit a negative response to louder background sound levels or compressor noise (Table 1) (Drosopoulos & Claridge, 2006; Greenfield, 2002; Hart, 2006; Hedwig, 2014). For example, the field cricket, *Gryllus bimaculatus*, uses acoustic signals during courtship and fails to orient toward male calls when exposed to traffic noise in a laboratory setting (Schmidt et al., 2014); yet, in our field investigation, the abundance of *Gryllus* was apparently unaffected by the presence of compressor noise or higher background sound levels. For those groups that are not affected by noise, some may have adjusted behaviors to cope with the increased sound level, others might not be sensitive to the particular frequencies of these noise sources (Morley et al., 2014), and some may experience other ecological influences that outweigh the potential effects of noise, such as reduced competition for limiting resources or decreases in predation.

Changes in arthropod abundances at the landscape scale could potentially result in a cascade of secondary ecological impacts via trophic interactions (van der Putten et al., 2004). Organisms that are the prey, predators, competitors, and beneficiaries of affected taxa might potentially experience auxiliary effects from noise‐induced changes on the abundances of these arthropods (sensu van der Putten et al., 2004). This study demonstrates the need for arthropod responses to be considered when ecosystems are exposed to noise pollution, and encourages future investigations to consider possible impacts of noise on multiple ecosystem components.

The lack of community turnover, despite indications of greater similarity within each site type (compressor and noncompressor), suggests that regardless of the effect of noise on some families and genera, the overall community composition at these two taxonomic levels is relatively stable. It is possible, however, that an examination at the species level would reveal a more nuanced response. Incomplete sampling, as demonstrated by the site‐specific rarefaction curves, might have limited our ability to detect site differences.

Due to the scale of noise exposure, and potential for covarying factors, like changes in vegetation structure, more data are needed to fully understand the relationship between arthropod communities and the acoustic environment. Future work should investigate these impacts at both the landscape and mechanistic levels. Landscape‐scale experiments could utilize playbacks to replicate compressor noise and other types of acoustic environments (natural and anthropogenic) and experimentally parse out the potential direct and indirect effects on arthropods. Laboratory experiments would aid in identifying the underlying mechanisms driving these ecologically important distributional patterns.

## Conflict of Interest

None declared.

## References

[ece32698-bib-0001] Akaike, H. (1974). A new look at the statistical model identification. IEEE Transactions on Automatic Control, AC‐19, 716–723.

[ece32698-bib-0002] Arnold, T. W. (2010). Uninformative parameters and model selection using Akaike's information criterion. Journal of Wildlife Management, 74, 1175–1178.

[ece32698-bib-0003] Barber, J. R. , Burdett, C. L. , Reed, S. E. , Warner, K. A. , Formichella, C. , Crooks, K. R. , ··· Fristrup, K. M. (2011). Anthropogenic noise exposure in protected natural areas: Estimating the scale of ecological consequences. Landscape Ecology, 26(9), 1281–1295.

[ece32698-bib-0004] Barber, J. R. , Crooks, K. R. , & Fristrup, K. M. (2010). The costs of chronic noise exposure for terrestrial organisms. Trends in Ecology & Evolution, 25(3), 180–189. 19762112 10.1016/j.tree.2009.08.002

[ece32698-bib-0005] Bayne, E. M. , Habib, L. , & Boutin, S. (2008). Impacts of chronic anthropogenic noise from energy‐sector activity on abundance of songbirds in the boreal forest. Conservation Biology, 22(5), 1186–1193. 18616740 10.1111/j.1523-1739.2008.00973.x

[ece32698-bib-0006] Berger, J. (2007). Fear, human shields and the redistribution of prey and predators in protected areas. Biology Letters, 3(6), 620–623. 17925272 10.1098/rsbl.2007.0415 PMC2391231

[ece32698-bib-0007] Bunkley, J. P. , McClure, C. J. W. , Kleist, N. J. , Francis, C. D. , & Barber, J. R. (2015). Anthropogenic noise alters bat activity levels and echolocation calls. Global Ecology and Conservation, 3, 62–71.

[ece32698-bib-0008] Claridge, M. F. (1985). Acoustic behavior of leafhoppers and planthoppers: Species problems and speciation In Nault L. R. & Rodriguez J. G. (Eds.), The leafhoppers and planthoppers (pp. 103–125). New York: Wiley.

[ece32698-bib-0009] Davies, T. W. , Bennie, J. , & Gaston, K. J. (2012). Street lighting changes the composition of invertebrate communities. Biology Letters. doi: 10.1098/rsbl.2012.0216 10.1098/rsbl.2012.0216 PMC3440964 22628095

[ece32698-bib-0010] Drosopoulos, S. , & Claridge, M. S. (2006). Insect Sounds and Communication: Physiology, Behaviour, Ecology and Evolution. Boca Raton: CRC Press Taylor & Francis Group.

[ece32698-bib-0011] Fassett, J. E. (2010) The San Juan Basin, a complex giant gas field, New Mexico and Colorado. Search and Discovery, Article #10254.

[ece32698-bib-0012] Francis, C. D. , & Barber, J. R. (2013). A framework for understanding noise impacts on wildlife: An urgent conservation priority. Frontiers in Ecology and the Environment, 11(6), 305–313.

[ece32698-bib-0013] Francis, C. D. , Kleist, N. J. , Ortega, C. P. , & Cruz, A. (2012). Noise pollution alters ecological services: Enhanced pollination and disrupted seed dispersal. Proceedings of the Royal Society B: Biological Sciences, 279, 2727–2735. 22438504 10.1098/rspb.2012.0230 PMC3367785

[ece32698-bib-0014] Francis, C. D. , Ortega, C. P. , & Cruz, A. (2009). Noise pollution changes avian communities and species interactions. Current Biology, 19(16), 1415–1419. 19631542 10.1016/j.cub.2009.06.052

[ece32698-bib-0015] Gibson, J. S. , & Uetz, G. W. (2008). Seismic communication and mate choice in wolf spiders: Components of male seismic signals and mating success. Animal Behaviour, 75(4), 1253–1262.

[ece32698-bib-0016] Gordon, S. D. , & Uetz, G. W. (2012). Environmental interference: Impact of acoustic noise on seismic communication and mating success. Behavioral Ecology, 23(4), 707–714.

[ece32698-bib-0017] Gotelli, N. J. , & Colwell, R. K. (2001). Quantifying biodiversity: Procedures and pitfalls in the measurement and comparison of species richness. Ecology Letters, 4(4), 379–391.

[ece32698-bib-0018] Gotelli, N. J. , & Colwell, R. K. (2011). Estimating species richness. Biological Diversity: Frontiers in Measurement and Assessment, 12, 39–54.

[ece32698-bib-0019] Gotelli, N. J. , & Ellison, A. M. (2004). A primer of ecological statistics. Sunderland: Sinauer Associates Inc.

[ece32698-bib-0020] Greenfield, M. D. (2002). Signalers and Receivers: Mechanisms and Evolution of Arthropod Communication. New York: Oxford University Press.

[ece32698-bib-0021] Hart, M. (2006). The role of sonic signals in the sexual communication of peach twig borers, Anarsia lineatella, Zeller (Lepidoptera: Gelechidae) [MS thesis]. Vancouver: Simon Fraser University.

[ece32698-bib-0022] Hedwig, B. (2014). Insect Hearing and Acoustic Communication. New York: Springer.

[ece32698-bib-0023] Hoy, R. R. , & Robert, D. (1996). Tympanal hearing in insects. Annual Review of Entomology, 41, 433–450. 10.1146/annurev.en.41.010196.002245 15012336

[ece32698-bib-0024] Jacobs, J. (2011). Individual Based Rarefaction using R‐package. Retrieved from http://www.jennajacobs.org/R/rarefaction.html

[ece32698-bib-0025] Lampe, U. , Schmoll, T. , Franzke, A. , & Reinhold, K. (2012). Staying tuned: Grasshoppers from noisy roadside habitats produce courtship signals with elevated frequency components. Functional Ecology, 26, 1348–1354.

[ece32698-bib-0026] Lee, Y. F. , & McCracken, G. F. (2005). Dietary variation of Brazilian free‐tailed bats links to migratory populations of pest insects. Journal of Mammalogy, 86(1), 67–76.

[ece32698-bib-0027] Mankin, R. W. (2012). Applications of acoustics in insect pest management. CAB Reviews, 7(1), 1–7. doi: 10.1079/PAVSNNR20127001

[ece32698-bib-0028] McIntyre, N. E. , Rango, J. , Fagan, W. F. , & Faeth, S. H. (2001). Ground arthropod community structure in a heterogeneous urban environment. Landscape and Urban Planning, 52(4), 257–274.

[ece32698-bib-0029] Mennitt, D. J. , & Fristrup, K. M. (2012). Obtaining calibrated sound pressure levels from consumer digital audio recorders. Applied Acoustics, 73, 1138–1145.

[ece32698-bib-0030] Meyer, J. , & Elsner, N. (1996). How well are frequency sensitivities of grasshopper ears tuned to species‐specific song spectra? Journal of Experimental Biology, 199, 1631–1642. 9319538 10.1242/jeb.199.7.1631

[ece32698-bib-0031] Morley, E. L. , Jones, G. , & Radford, A. N. (2014). The importance of invertebrates when considering the impacts of anthropogenic noise. Proceedings of the Royal Society B, 281, 20132683. 24335986 10.1098/rspb.2013.2683 PMC3871318

[ece32698-bib-0032] Nowacek, D. P. , Thorne, L. H. , Johnston, D. W. , & Tyack, P. L. (2007). Responses of cetaceans to anthropogenic noise. Mammal Review, 37(2), 81–115.

[ece32698-bib-0033] Ohwaki, A. (2015). Ground arthropod communities in paddy fields during the dry period: Comparison between different farming methods. Journal of Asia‐Pacific Entomology, 18(3), 413–419.

[ece32698-bib-0034] Polidori, C. , Pavan, G. , Ruffato, G. , Asis, J. D. , & Tormos, J. (2013). Common features and species‐specific differences in stridulatory organs and stridulation patterns of velvet ants (Hymenoptera: Mutillidae). Zoologischer Anzeiger, 252, 457–468.

[ece32698-bib-0035] Prather, C. M. , Shannon, L. P. , Laws, A. , Rivest, E. , Woltz, M. , Bloch, C. P. , ··· Joern, A. (2012). Invertebrates, ecosystem services and climate change. Biological Reviews, 88, 328–348. 10.1111/brv.12002 23217156

[ece32698-bib-0036] van der Putten, W. H. , de Ruiter, P. C. , Bezemer, T. M. , Harvey, J. A. , Wassen, M. , & Wolters, V. (2004). Trophic interactions in a changing world. Basic and Applied Ecology, 5(6), 487–494.

[ece32698-bib-0037] R Development Core Team (2015). R: A language and environment for statistical computing. Vienna, Austria: R Foundation for Statistical Computing http://www.R-project.org.

[ece32698-bib-0038] Saxena, K. N. , & Kumar, H. (1980). Interruption of acoustic communication and mating in a leafhopper and a planthopper by aerial sound vibrations picked up by plants. Experientia, 36(8), 933–936.

[ece32698-bib-0039] Schmidt, R. , Morrison, A. , & Kunc, H. P. (2014). Sexy voices –‐ no choices: Male song in noise fails to attract females. Animal Behavior, 94, 135–141.

[ece32698-bib-0040] Shamble, P. S. , Menda, G. , Golden, J. R. , Nitzany, E. I. , Walden, K. , Beatus, T. , ··· Hoy, R. R. (2016). Airborne acoustic perception by a jumping spider. Current Biology, 26, 2913–2920. 27746028 10.1016/j.cub.2016.08.041 PMC5102792

[ece32698-bib-0041] Shieh, B. S. , Liang, S. H. , Chen, C. C. , Loa, H. H. , & Liao, C. Y. (2012). Acoustic adaptations to anthropogenic noise in the cicada *Cryptotympana takasagona* Kato (Hemiptera: Cicadidae). Acta Ethologica, 15, 33–38.

[ece32698-bib-0042] Slabbekoorn, H. , Bouton, N. , van Opzeeland, I. , Coers, A. , ten Cate, C. , & Popper, A. N. (2010). A noisy spring: The impact of globally rising underwater sound levels on fish. Trends in Ecology & Evolution, 25(7), 419–427. 20483503 10.1016/j.tree.2010.04.005

[ece32698-bib-0043] Spence, J. R. , & Niemela, J. K. (1994). Sampling carabid assemblages with pitfall traps: The madness and the method. The Canadian Entomologist, 126, 881–894.

[ece32698-bib-0044] Stritih, N. , & Cokl, A. (2012). Mating behaviour and vibratory signaling in the non‐hearing cave crickets reflect primitive communication of ensifera. PLoS One, 7(10), e47646. 23094071 10.1371/journal.pone.0047646 PMC3477131

[ece32698-bib-0045] Tishechkin, D. Y. (2003). Vibrational communication in Cercopoidea and Fulgoroidea (Homoptera: Cicadina) with notes on classification of higher taxa. Russian Entomological Journal, 12(2), 129–181.

