# Supplementary material for: Anthropogenic noise changes arthropod abundances
Source: Ecol Evol. 2017 Mar 23;7(9):2977–85. doi: 10.1002/ece3.2698 (PMC5415529; doi:10.1002/ece3.2698)
Supplement: Supplementary file 1 [file ECE3-7-2977-s001.docx]

**Appendix**


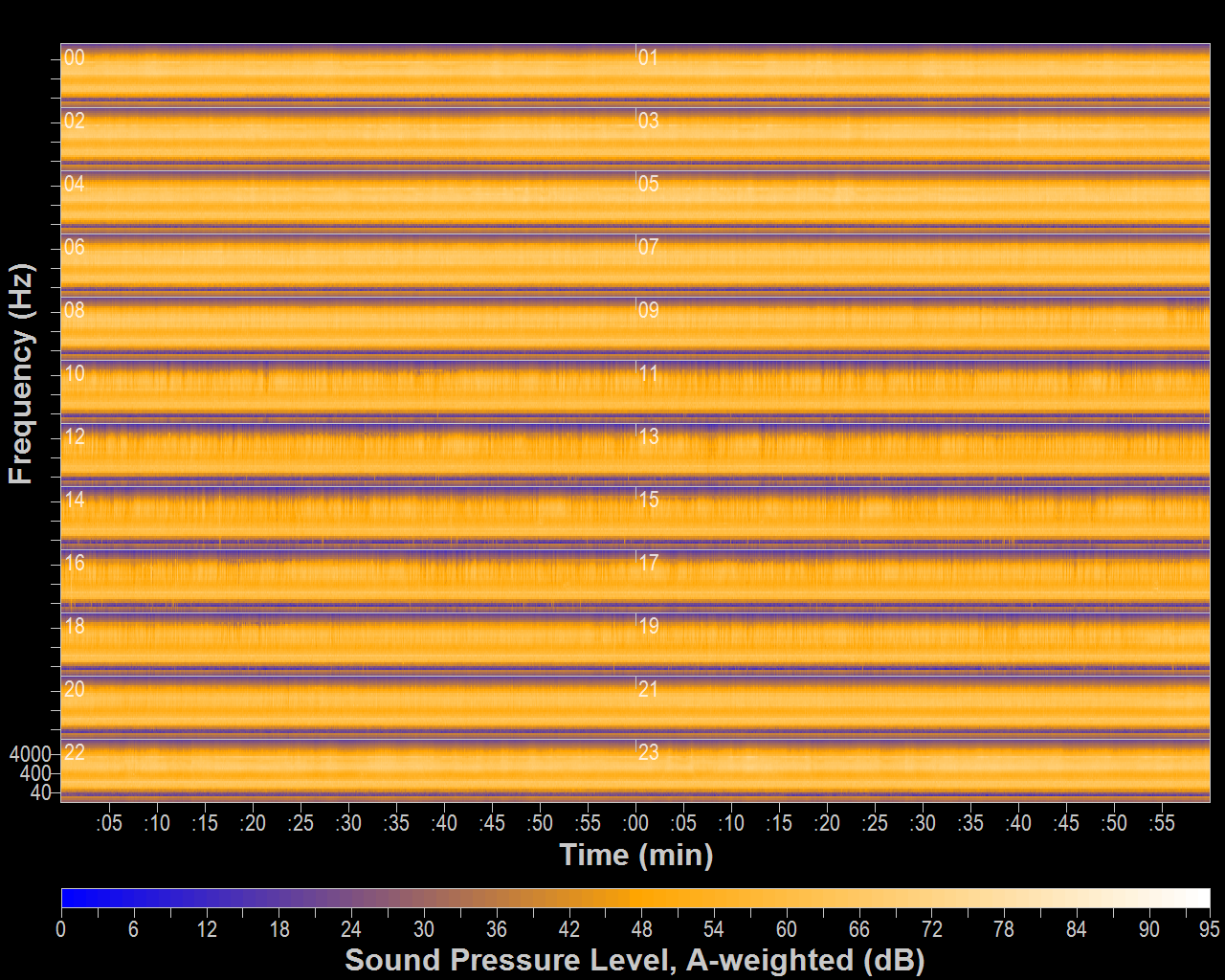
_
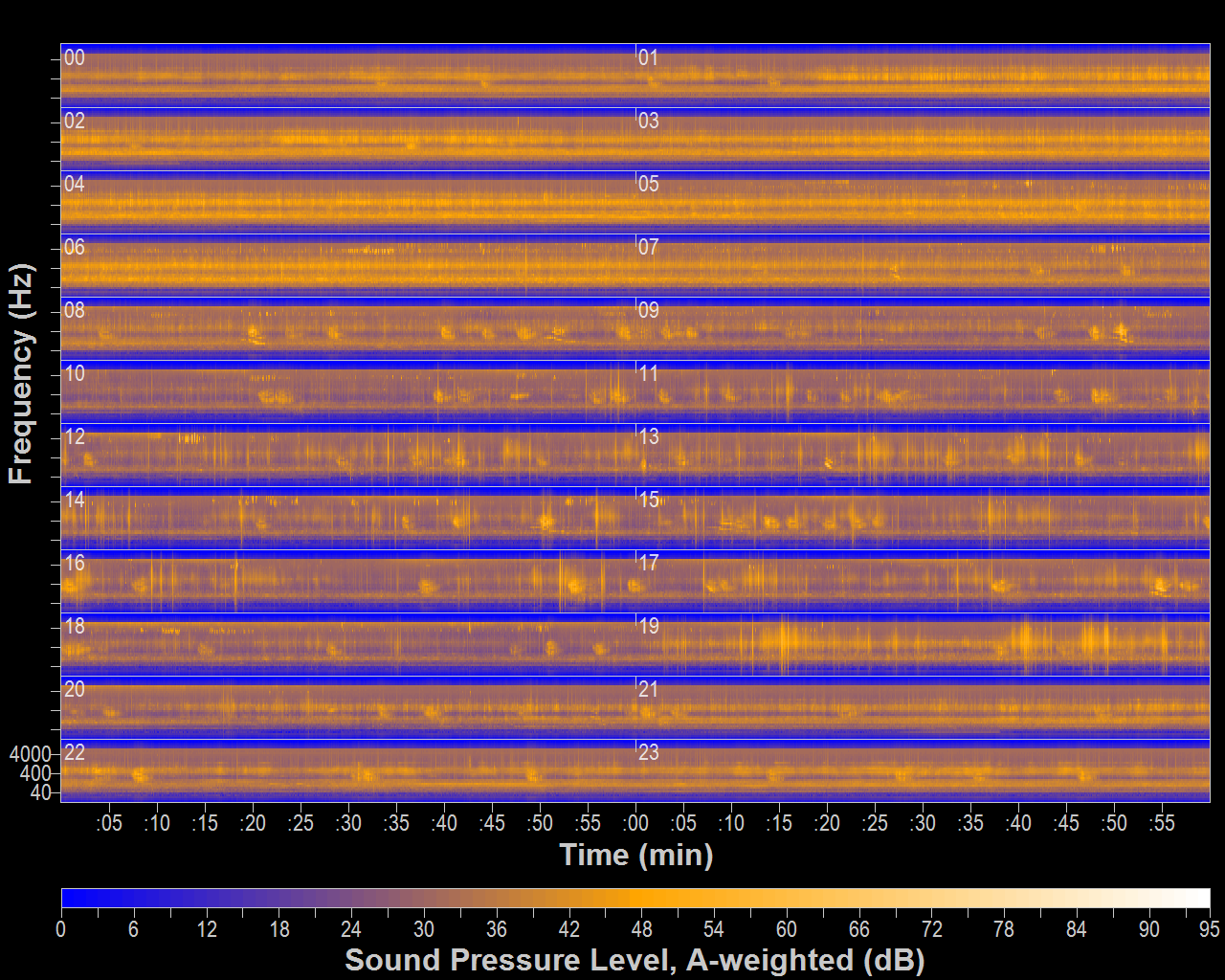
_

S1. Twenty-four hour spectrograms of a non-compressor site (left) and compressor site (right) made using Roland R05 recording units, MP3 128 kbps. Frequency (kHz) is on the y-axis and time (minutes) is on the x-axis. Each line represents a two-hour block of time. The intensity of the color ramp illustrates sound amplitude or volume. There are a variety of sounds present at the non-compressor site, including some apparent anthropogenic noise in the first eight hours and biological sounds in the subsequent 16 hours.

S2. Background sound levels (dB A) and site type for each sampling location.

| **Sampling Location** | **Site Type** | **dB(A)** |
| --- | --- | --- |
| 1 | Non-Compressor | 54.9 |
| 2 | Non-Compressor | 58.2 |
| 3 | Non-Compressor | 62.2 |
| 4 | Non-Compressor | 66.9 |
| 5 | Non-Compressor | 68.8 |
| 6 | Compressor | 68.9 |
| 7 | Compressor | 72.2 |
| 8 | Compressor | 75.4 |
| 9 | Compressor | 76.6 |
| 10 | Compressor | 80.8 |


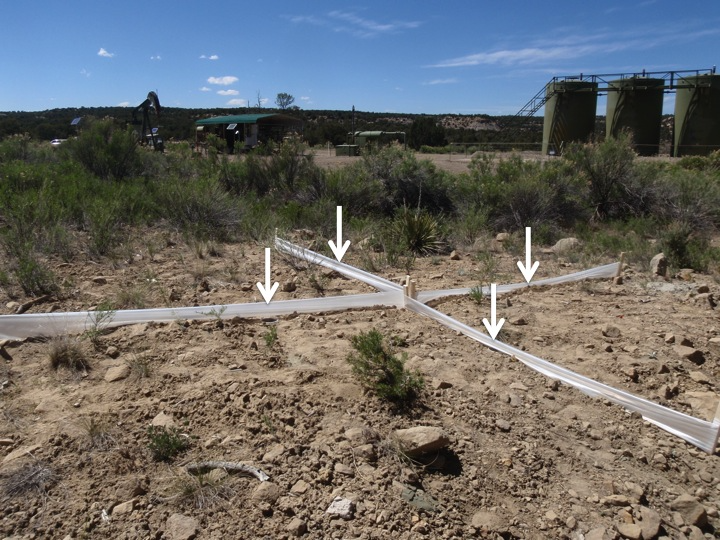


S3. Trap arrangement at a site with a compressor station. The four pitfall traps (identified with white arrows) were arranged in an X grid with plastic guide vanes positioned perpendicular to the traps.

S4. Table of sample variances (rows) for each sampling location (columns) (program R). Sampling location numbers correspond to those is A2 (locations 1-5 are non-compressor sites and 6-10 are compressor sites).

|  | **1** | **2** | **3** | **4** | **5** | **6** | **7** | **8** | **9** | **10** |
| --- | --- | --- | --- | --- | --- | --- | --- | --- | --- | --- |
| **1** | 8.53 | 3.77 | 7.02 | 131.49 | 2.75 | 144.04 | 1886.73 | 32.73 | 1.92 | 1.56 |
| **2** | 1.94 | 2.56 | 2.00 | 17.06 | 0.79 | 17.92 | 22.33 | 1.98 | 1.06 | 1.67 |
| **3** | 4.59 | 2.04 | 2.43 | 3.25 | 2.48 | 10.57 | 14.36 | 0.64 | 0.60 | 6.22 |
| **4** | 1.30 | 3.36 | 3.37 | 6.75 | 1.86 | 5.84 | 21.86 | 4.53 | 0.19 | 1.55 |
| **5** | 3.52 | 0.42 | 6.76 | 5.76 | 0.99 | 6.51 | 15.73 | 1.96 | 0.92 | 3.05 |
| **6** | 0.40 | 1.22 | 10.76 | 27.29 | 1.80 | 13.21 | 5.13 | 1.03 | 1.42 | 2.54 |
| **7** | 0.73 | 0.44 | 2.14 | 39.26 | 1.44 | 8.73 | 3.93 | 1.60 | 2.24 | 0.97 |
| **Average** | 3.00 | 1.97 | 4.93 | 32.98 | 1.73 | 29.55 | 281.44 | 6.35 | 1.20 | 2.51 |

S5. The AIC tables, β, and Standard Errors of the arthropod families and nested genera where the compressor or dB variable was in the top model. Those groups with an * are significant at the 95% confidence level (strong evidence) and all others are significant at the 85% confidence level (marginal evidence).

| **Family:** | **Acrididae *** | |  |  |  |  |
| --- | --- | --- | --- | --- | --- | --- |
| **Model** | **K** | **AIC** | **∆AIC** | **Wi** | **β** | **SE** |
| Compressor | 3 | 236.06 | 0.00 | 0.40 | -0.47 | 0.29 |
| Null | 2 | 236.36 | 0.30 | 0.35 |  |  |
| dB | 3 | 237.00 | 0.94 | 0.25 | -0.02 | 0.02 |
|  |  |  |  |  |  |  |
| **Family:** | **Cercopidae** | |  |  |  |  |
| **Model** | **K** | **AIC** | **∆AIC** | **Wi** | **β** | **SE** |
| Compressor | 3 | 101.95 | 0.00 | 0.54 | -1.17 | 0.63 |
| Null | 2 | 103.01 | 1.06 | 0.32 |  |  |
| dB | 3 | 104.69 | 2.74 | 0.14 | -0.03 | 0.05 |
|  |  |  |  |  |  |  |
| **Family:** | **Cicadellidae *** | |  |  |  |  |
| **Model** | **K** | **AIC** | **∆AIC** | **Wi** | **β** | **SE** |
| dB | 3 | 296.16 | 0.00 | 0.48 | 0.36 | 0.21 |
| Null | 2 | 296.87 | 0.70 | 0.34 |  |  |
| Compressor | 3 | 298.21 | 2.05 | 0.17 | 0.36 | 0.44 |
|  |  |  |  |  |  |  |
| **Family:** | **Lycosidae *** | |  |  |  |  |
| **Model** | **K** | **AIC** | **∆AIC** | **Wi** | **β** | **SE** |
| dB | 3 | 89.00 | 0.00 | 0.57 | -0.58 | 0.26 |
| Compressor | 3 | 90.75 | 1.75 | 0.24 | -0.94 | 0.59 |
| Null | 2 | 91.13 | 2.13 | 0.20 |  |  |
|  |  |  |  |  |  |  |
|  | **Genus: *Pardosa* *** | |  |  |  |  |
| **Model** | **K** | **AIC** | **∆AIC** | **Wi** | **β** | **SE** |
| dB | 3 | 72.64 | 0.00 | 0.60 | -0.75 | 0.01 |
| Compressor | 3 | 74.73 | 2.10 | 0.21 | -1.16 | 0.77 |
| Null | 2 | 74.91 | 2.27 | 0.19 |  |  |
|  |  |  |  |  |  |  |
| **Family:** | **Mutillidae *** | |  |  |  |  |
| **Model** | **K** | **AIC** | **∆AIC** | **Wi** | **β** | **SE** |
| dB | 3 | 69.07 | 0.00 | 0.85 | -0.82 | 0.31 |
| Compressor | 3 | 73.18 | 4.11 | 0.11 | -1.20 | 0.66 |
| Null | 2 | 74.87 | 5.80 | 0.05 |  |  |
|  |  |  |  |  |  |  |
| **Family:** | **Rhaphidophoridae, Genus: *Ceuthophilus **** | | | | | |
| **Model** | **K** | **AIC** | **∆AIC** | **Wi** | **β** | **SE** |
| Compressor | 3 | 102.37 | 0.00 | 0.69 | -3.66 | 1.55 |
| dB | 3 | 104.79 | 2.43 | 0.21 | -0.18 | 0.09 |
| Null | 2 | 106.20 | 3.83 | 0.10 |  |  |

S6. The AIC tables, β, and Standard Errors of the arthropod families and nested genera where the compressor or dB variable were not in the top model.

| **Family:** | **Chrysomelidae** | | |  |  |  |
| --- | --- | --- | --- | --- | --- | --- |
| **Model** | **K** | **AIC** | **∆AIC** | **Wi** | **β** | **SE** |
| Null | 2 | 73.99 | 0.00 | 0.58 |  |  |
| Compressor | 3 | 75.99 | 2.00 | 0.21 | 0.04 | 1.00 |
| dB | 3 | 75.99 | 2.00 | 0.21 | -0.00 | 0.07 |
|  |  |  |  |  |  |  |
| **Family:** | **Formicidae** | |  |  |  |  |
| **Model** | **K** | **AIC** | **∆AIC** | **Wi** | **β** | **SE** |
| Null | 2 | 912.73 | 0.00 | 0.56 |  |  |
| Compressor | 3 | 914.56 | 1.83 | 0.22 | 0.22 | 0.53 |
| dB | 3 | 914.67 | 1.95 | 0.21 | -0.00 | 0.03 |
|  |  |  |  |  |  |  |
| **Family:** | **Gnaphosidae** | |  |  |  |  |
| **Model** | **K** | **AIC** | **∆AIC** | **Wi** | **β** | **SE** |
| Null | 2 | 247.74 | 0.00 | 0.54 |  |  |
| Compressor | 3 | 249.46 | 1.72 | 0.23 | 0.28 | 0.54 |
| dB | 3 | 249.49 | 1.75 | 0.23 | -0.02 | 0.04 |
|  |  |  |  |  |  |  |
| **Family:** | **Gryllidae** | |  |  |  |  |
| **Model** | **K** | **AIC** | **∆AIC** | **Wi** | **β** | **SE** |
| Null | 2 | 80.26 | 0.00 | 0.57 |  |  |
| Compressor | 3 | 82.15 | 1.89 | 0.22 | -0.28 | 0.83 |
| dB | 3 | 82.20 | 1.94 | 0.21 | -0.01 | 0.05 |
|  |  |  |  |  |  |  |
| **Family:** | **Salticidae** | |  |  |  |  |
| **Model** | **K** | **AIC** | **∆AIC** | **Wi** | **β** | **SE** |
| Null | 2 | 81.35 | 0.00 | 0.54 |  |  |
| Compressor | 3 | 82.87 | 1.52 | 0.25 | -0.40 | 0.57 |
| dB | 3 | 83.33 | 1.99 | 0.20 | 0.00 | 0.04 |
|  |  |  |  |  |  |  |
| **Family:** | **Tenebrionidae** | | |  |  |  |
| **Model** | **K** | **AIC** | **∆AIC** | **Wi** | **β** | **SE** |
| Null | 2 | 119.40 | 0.00 | 0.47 |  |  |
| Compressor | 3 | 120.00 | 0.60 | 0.35 | 0.47 | 0.40 |
| dB | 3 | 121.21 | 1.81 | 0.19 | 0.01 | 0.03 |
|  |  |  |  |  |  |  |
| **Genus:** | ***Spharagemon*** | | |  |  |  |
| **Model** | **K** | **AIC** | **∆AIC** | **Wi** | **β** | **SE** |
| Null | 2 | 105.53 | 0.00 | 0.52 |  |  |
| dB | 3 | 106.92 | 1.39 | 0.26 | 0.06 | 0.08 |
| Compressor | 3 | 107.33 | 1.80 | 0.21 | 0.49 | 1.14 |
|  |  |  |  |  |  |  |
| **Genus:** | ***Xanthippus*** | |  |  |  |  |
| **Model** | **K** | **AIC** | **∆AIC** | **Wi** | **β** | **SE** |
| Null | 2 | 98.18 | 0.00 | 0.55 |  |  |
| Compressor | 3 | 99.76 | 1.58 | 0.25 | -0.39 | 0.59 |
| dB | 3 | 100.14 | 1.96 | 0.21 | -0.01 | 0.04 |

1.
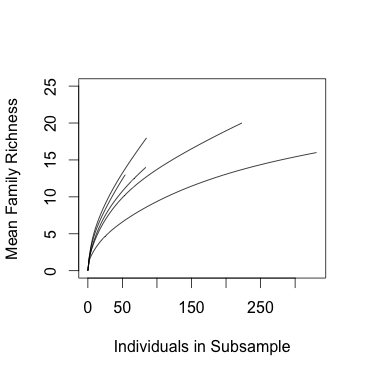

2.
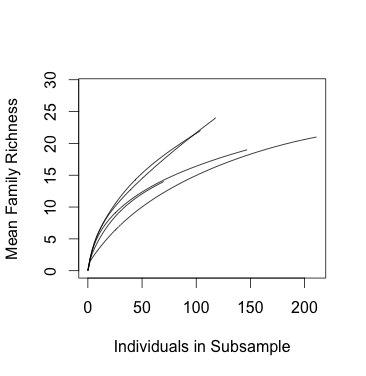


S7. Site specific rarefaction curves for A) compressor sites (dashed vertical line indicates the smallest number of individuals (54) sampled at any site) and B) non-compressor sites.


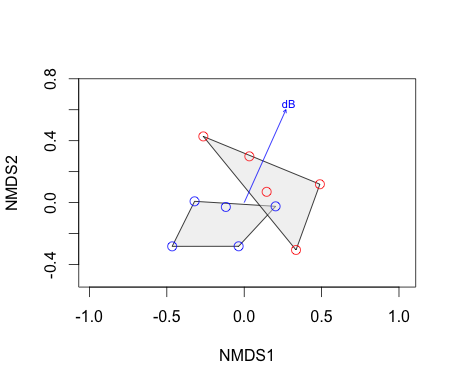


S8. The Non-Metric Multidimensional Scaling plot shows the grouping of non-compressor sites (blue circles) and compressor sites (red circles), with slight overlap. A post-hoc fit of the dB(A) environmental vector confirms the relationship between the presence of a compressor and background sound level.


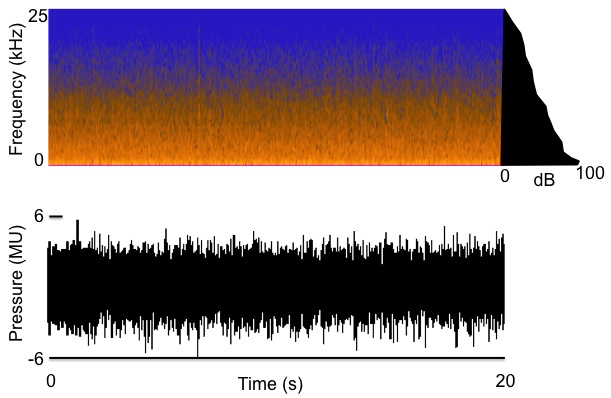


S9. Visualization of compressor noise. The spectrogram shows the frequencies on the Y-axis and time on the X-axis and the power spectra to the right indicates the relative intensities (dB, X-axis) of each frequency (Y-axis). The sound wave is represented by an oscillogram beneath the spectrogram.
